# Supplementary material for: Weighted Burden Analysis of Exome-Sequenced Case-Control Sample Implicates Synaptic Genes in Schizophrenia Aetiology
Source: Behav Genet. 2018 Mar 21;48(3):198–208. doi: 10.1007/s10519-018-9893-3 (PMC5934462; doi:10.1007/s10519-018-9893-3)
Supplement: Supplementary file 1 — Supplementary material 1 (DOCX 15 KB) [file 10519_2018_9893_MOESM1_ESM.docx]

**Weighted burden analysis of exome-sequenced case-control sample implicates synaptic genes in schizophrenia aetiology**

**Supplementary table S1**

The table shows the weight accorded to each type of variant as annotated by VEP (McLaren et al., 2016). 10 was added to this weight if the variant was annotated by Polyphen as possibly or probably damaging and 10 was added if SIFT annotated it as deleterious (Adzhubei et al., 2013; Kumar et al., 2009).

| VEP annotation | Weight |
| --- | --- |
| intergenic_variant | 1 |
| feature_truncation | 3 |
| regulatory_region_variant | 3 |
| feature_elongation | 3 |
| regulatory_region_amplification | 3 |
| regulatory_region_ablation | 3 |
| TF_binding_site_variant | 3 |
| TFBS_amplification | 3 |
| TFBS_ablation | 3 |
| downstream_gene_variant | 3 |
| upstream_gene_variant | 3 |
| non_coding_transcript_variant | 3 |
| NMD_transcript_variant | 3 |
| intron_variant | 3 |
| non_coding_transcript_exon_variant | 3 |
| 3_prime_UTR_variant | 5 |
| 5_prime_UTR_variant | 5 |
| mature_miRNA_variant | 5 |
| coding_sequence_variant | 5 |
| synonymous_variant | 5 |
| stop_retained_variant | 5 |
| incomplete_terminal_codon_variant | 5 |
| splice_region_variant | 5 |
| protein_altering_variant | 10 |
| missense_variant | 10 |
| inframe_deletion | 15 |
| inframe_insertion | 15 |
| transcript_amplification | 15 |
| start_lost | 15 |
| stop_lost | 15 |
| frameshift_variant | 20 |
| stop_gained | 20 |
| splice_donor_variant | 20 |
| splice_acceptor_variant | 20 |
| transcript_ablation | 20 |
